# Incremental Yield of Serial Sputum Cultures for Diagnosis of Tuberculosis among HIV Infected Smear Negative Pulmonary TB Suspects in Kampala, Uganda

**DOI:** 10.1371/journal.pone.0037650

**Published:** 2012-05-22

**Authors:** Willy Ssengooba, Noah Kiwanuka, David P. Kateete, Achilles Katamba, Moses L. Joloba

**Affiliations:** 1 School of Biomedical Sciences, Makerere University College of Health Sciences, Kampala, Uganda; 2 School of Public Health, Makerere University College of Health Sciences, Kampala, Uganda; 3 School of Medicine, Makerere University College of Health Sciences, Kampala, Uganda; National Institute of Infectious Diseases, Japan

## Abstract

**Background:**

Sputum culture is the gold standard for diagnosis of pulmonary tuberculosis (PTB). Although mostly used for research, culture is recommended by the World Health Organization for TB diagnosis among HIV infected smear negative PTB suspects. Even then, the number of sputum samples required remains unspecified. Here, we determined the Incremental Yield (IY) and number of samples required to diagnose an additional PTB case upon second and third serial sputum culture.

**Methods/Findings:**

This was a cross sectional study done between January and March 2011. Serial sputum samples were provided by participants within two days and cultured using Lowenstein Jensen (LJ) and Mycobacteria Growth Indicator Tube (MGIT) methods. A PTB case was defined as a positive culture on either one or both methods. The IY from the second and third serial cultures was determined and the reciprocal of the product of the fractions of IY provided the number of samples required for an additional PTB case. Of the 170 smear negative PTB suspects, 62 (36.5%) met the case definition. The IY of the second sample culture was 12.7%, 23.6% and 12.6% and for the third sample culture was 6.8%, 7.5% and 7.3% with LJ, MGIT and LJ or MGIT, respectively. The number of samples required for an additional PTB case and 95% CI upon the second sample culture were 29.9 (16.6, 156.5), 11.3 (7.6, 21.9) and 20.8 (12.5, 62.7); while for the third sample culture were 55.6 (26.4, 500.4), 35.7 (19.0, 313.8) and 36.1 (19.1, 330.9) by LJ, MGIT and LJ or MGIT respectively.

**Conclusions/Significance:**

Among HIV infected smear negative PTB suspects in Kampala, 93% of PTB cases are diagnosed upon the second serial sputum culture. The number of cultures needed to diagnose an additional PTB case, ranges from 11–30 and 35–56 by the second and third sputum samples, respectively.

## Introduction

SPUTUM CULTURE remains the gold standard for diagnosis of pulmonary tuberculosis (PTB). Although mainly used for research, culture is recommended by the World Health Organization (WHO) for TB diagnosis among HIV-infected smear negative PTB suspects [1].

Several studies have documented the usefulness of sputum culture among HIV-infected smear negative TB suspects. Smear negative, culture positive TB prevalence ranged from 32% to 56% [2], [3], [4]. However, the number of samples required for sputum culture remains unspecified despite the resource constraints. There is only one public TB culture facility in Uganda, where culture is mainly done to rule out drug resistant TB. There have been advances in decentralizing sputum culture services so that they are more accessible; however, evidence-based means and best practices towards their efficient use have not been fully explored [5].

Some of the limitations towards realized access to the existing TB culture facilities are the operational costs. One of the ways to reduce this gap could be determining the optimal number of sputum samples to be cultured. There are variations in the number of sputum samples submitted for culture as a result of trying a trade-off between cost and increase in TB case detection [6], [7], [8].

Reducing the number of sputum specimens and clinic visits for diagnosis in settings with high prevalence of TB and HIV could reduce resources needed and workload for laboratory staff, with improved compliance to sputum sample collection procedures [9].

In this current study, we determined the Incremental Yield (IY) and the number of sputum samples required for an additional PTB case upon second and third serial sputum sample culture among HIV infected smear negative PTB suspects in order to inform practice and efficient use of the available TB culture resources.

## Results

Out of the 203 PTB suspects, 22 (10.8%) did not consent and 11 (5.4%) turned positive on repeated smear microscopy. We analyzed results from 170 (84.0%) smear negative PTB suspects and their characteristics are summarized in Table 1. Among the eligible participants, 104 (61.2) were in WHO HIV/AIDS clinical stage 2, 36 (21.2%) were in stage 3, 30 (17.6%) were in stage 1 and 86(50.6%) of the participants were on Anti Retroviral Therapy (ART).

**Table 1:** Prevalence of pulmonary tuberculosis (PTB) among sputum smear negative HIV infected TB suspects (n = 170).

Category	Parameter	Culture positive/TB cases (n = 62)	Culture negative (n = 108)
**Gender**	Females	34 (54.8%)	68 (63.0%)
	Males	28 (45.2%)	40 (37.0%) P = 0.285
**Age**	Mean (SD)	36.5(9.4)	
	Median (IQR)	35(32–37)	
**Age groups (Years)**	20–30	21 (33.9%)	31(28.7%)
	31–40	24 (38.7%)	45(41.7%)
	41–50	13 (21.0%)	21(19.4%)
	51–60	4 (6.5%)	7(6.5%)
	61–70	0 (0.0%)	2(1.9%)
	Unknown	0 (0.0%)	2(1.9%)

SD = Standard Deviation, IQR = Inter Quartile Range.

Of the 170 participants, 20 (11.7%) failed to submit the second and/or the third sputum sample; thus, a total of 461 (90.4%) sputum samples were analyzed. Sixty-nine (16.4%) became contaminated upon MGIT culture, of which 25 (36.2%) were first, 35 (50.7%) second and nine (13.1%) third sputum samples. A contamination rate of 2% was documented on LJ. All contaminated samples were categorized as negative during the analysis, Table 1.

Of the 170 participants, 43 (25.3%), 60 (35.3%) and 62 (36.5%) were culture positive for MTBC using LJ alone, MGIT alone and LJ or MGIT, respectively.

Out of the 62 culture positive participants, four (2.4%) had both MTBC and *Mycobacterium* Other Than Tuberculosis (MOTT) hence met the TB case definition. Females and males were equally likely to be culture positive, p = 0.285, Table 1.

All participants who submitted at least one sample for culture were considered in the analysis while catering for the probability of those samples missed being positive when the previously cultured sample (s) were negative. Majority of the PTB cases were detected on the first and less were detected by the third sputum sample culture. Table 2 shows the results obtained by sputum culture in six different patterns according to sample number.

**Table 2:** Distribution of sputum sample culture results for the six patterns of results according to methods used.

Pattern of result	LJ alone n (%)	MGIT alone n (%)	LJ or MGIT n (%)
**Px**	36 (21.2)	44 (25.9)	52 (30.6)
**NPx**	5 (2.9)	13 (7.7)	7 (4.2)
**NNP**	2 (1.2)	3 (1.7)	3 (1.7)
**NNN**	82 (48.2)	67 (39.4)	67 (39.4)
**NN9**	29 (17.1)	26 (15.3)	24 (14.1)
**N99**	16 (9.4)	17 (10.0)	17 (10)
**Total patterns/patients:**	**170**	**170**	**170**

P_X_ = positive on the first sample, **x** = the next result adds no more diagnostic value, **N** = Negative, 9 = missing, LJ (Solid culture) = Lowenstein Jensen, MGIT (Liquid culture) = Mycobacterium Growth Indicator Tube.

The patterns from table 2 were used to determine the IY for the second and third sputum samples for each culture method and when both methods were combined.

The IY of the second and third sputum sample culture were 12.7% and 6.8% on LJ, 23.6% and 7.5% on MGIT and 12.6% and 7.3% on LJ or MGIT culture methods respectively, Table 3. Therefore, on average, 80%, 13%, and 7% of the TB cases where detected on the first third sputum sample cultures respectively. This indicates that 93% of the TB cases are detected upon a second sputum sample culture.

**Table 3:** Yield of first and Incremental yield of the second and third serial sputum culture.

Observed and expected incrementalyield	LJ	MGIT	LJ orMGIT
Observed yield from 1st sputum culture:	0.212	0.259	0.306
Observed yield from 2nd sputum culture:	0.042	0.119	0.069
Observed yield from 3rd sputum culture:	0.024	0.043	0.043
Total positives observed	43	60	62
Missed TB cases %¥			
Positives missed by not doing a 2nd sputum culture	0.7	2.0	1.2
Positives missed by not doing a 3rd sputum culture	1.1	1.8	1.7
Total positives missed	1.8	3.8	2.9
Expected yield from 3 sputum cultures (Exp)π	44.8	63.8	64.9
Expected Yield (Fraction)†			
Observed pattern (n) + missed TB cases/Exp	1.000	1.000	1.000
Expected from the first sputum culture‡_1_	0.805	0.690	0.801
Expected from second sputum culture ‡_2_	0.127	0.236	0.126
Expected from third sputum culture‡_3_	0.068	0.075	0.073

LJ (Solid culture) = Lowenstein Jensen, MGIT (Liquid culture) = Mycobacterium Growth Indicator Tube.

¥ = Number of estimated TB cases missed among those who never submitted the 2^nd^ and 3^rd^ sputum sample for culture, assuming a same proportion of TB cases as in those who submitted the 2^nd^ and 3^rd^ sputum for culture, respectively. † = The proportion of TB cases expected if each of the suspects had a complete set of three sputum cultures, *Π* = Total positives observed + total positives missed, ‡_1_ = Px/Exp, ‡_2_ = NPx + missed by second/ExP and ‡_3_ = NNP + missed by third/Exp.

The number of samples needed to find one additional case and their 95% CI by the second and third sputum culture for LJ was 29.9 (16.6, 156.5), 55.6 (26.4, 500.4), MGIT11.3 (7.6, 21.9), 35.7 (19.0, 313.8) and using LJ or MGIT 20.8 (12.5, 62.7) and 36.1 (19.1, 330.9) respectively. These were compared with the maximum number from the TB expert opinions taken as the critical values, Table 4.

**Table 4:** Comparison of Number of sputum samples required to detect one additional TB case on 2^nd^ and 3^rd^ sputum culture versus TB expert critical values.

Culture method	First sample culture (spot specimen)n (95% CI)^a^	Second sample culture (morning specimen)n (95% CI)^b^	Expert opinion on2nd sample culture	Third sample culture (spot specimen)n (95% CI)^c^	Expert opinion on3rd sample culture
**LJ alone**	4.7 (3.7–6.7)	29.9 (16.6–156.5)	2^1^, 3^2,3^, 10^4^, 12 **µ** ^ 5^	55.6 (26.4–500.4)	3^2,3^, 5^1^, 17 **µ** ^ 5^
**MGIT alone**	3.9 (3.1–5.2)	**11.3** (7.6–21.9)	2^1^, 3^2,3^, 10^4^, **12** **µ** **^5^**	35.7 (19.0–313.8)	3^2,3^, 5^1^, 17 **µ** ^ 5^
**LJ plus MGIT**	3.3 (2.7–4.2)	20.8 (12.5–62.7)	2^1^, 3^2,3^, 10^4^, 12 **µ** ^ 5^	36.1 (19.1–330.9)	3^2,3^, 5^1^, 17 **µ** ^ 5^

LJ (Solid culture) = Lowenstein Jensen, MGIT (Liquid culture) = Mycobacterium Growth Indicator Tube.

1 Derek Armstrong, ^2^Richard Oatis, ^3^John Kenneth ^4∼^Armand Van Deun, ^5^Nicole Parrish;

Order has no superiority meaning.

∼ = the third sample was not necessary and could not recommend for it to be examined.

CI = Confidence Interval.

a _= _1/Px*170, **^b^** = 1/NPx +positive missed by second*170 and **^c^** = 1/NNP + positive missed by third * 170, **µ** = Expert opinion Critical value for the second and third sputum sample culture.

## Discussion

This cross-sectional study reveals that among HIV-infected smear negative PTB suspects in Kampala Uganda, the prevalence of PTB was 36.5%. More than 93% of these cases were diagnosed with two serial sputum sample culture. The number of sputum samples needed to be examined to identify an additional PTB case ranged from 11–30 samples, by the second and 35 –56 samples by the third sputum sample culture. The number of samples by the 3rd sputum culture exceeded what the TB experts suggested as acceptable maximum number i.e. 17 samples.

Previous studies done among HIV infected patients documented similar PTB prevalence among smear negative TB suspects; a study done Thailand and Vietnam showed a PTB prevalence of 32% [2] and in Zimbabwe a prevalence of 39.4% was documented [4]. However a study done in Uganda [3] documented PTB prevalence of 56%, probably it considered only admitted patients who were more likely to have more cavities than the participants in the previous studies as well as this study.

In HIV symptomatic patients with at least two negative sputum smears and one culture negative, it is important to question if culture is more helpful than clinical evaluations and if it is worth the effort to ask for a second culture if the physician is still considering TB after all those negative results.

Using LJ, less numbers of PTB cases were detected; 43 (69.3%) compared to MGIT method; 60 (96.7%). This difference is mainly due to the fact that MGIT, which is a liquid culture system, is more sensitive but also more prone to contamination than LJ method, which is a solid culture system, as also documented in other studies [2], [10], [11]. The differences in proportions of PTB cases found in this population using different culture methods among different sputum sample cultures suggest that locally designed studies are needed to determine the value of the serial sputum samples [12], [13]. The high contamination rate of MGIT culture, not only increase the cost for culture, but also leads to under estimation of prevalence of PTB obtained from MGIT method as all contaminated cultures were classified as negative during the analysis.

The IY of the second and third sputum sample culture documented by this study also agrees with the previous studies. A study conducted in Thailand and Vietnam among HIV infected persons [2] documented an IY by LJ method of 14%; 95% CI 9–22, and 6%; 95% CI 3–12) for the second and third sputum sample cultures respectively as compared to IY by MGIT method of 17%; 95% CI 11–24 and IY 10%; 95%CI5–16, for the second and third sputum cultures respectively. However, David Moore et al documented minimal gain (only in IY) from the second sputum sample culture and did not consider a third sputum sample culture [14]. These studies, however, did not analyze the IY for LJ or MGIT which is a common practice in some countries. The increased yield of the second MGIT culture could be partly due to the second culture detecting the first contaminated cultures; however, contamination is unlikely to fully explain the IY in MGIT because this increased yield was also observed by other studies with lower contamination rates [2], [14].

Furthermore, previous studies have urged that more than two sputum samples are not worthy being examined comparing the diagnostic gain vis-à-vis the burden to scarcity of human resource, financial constraints, limited laboratory facilities and high workload, which could in turn affect the quality of the results obtained from the third sputum sample examination [12], [6].

Although these studies were not using sputum culture and were done in both HIV infected and non-HIV infected patients, they are in agreement with this study as the incremental yield from the third sputum sample for resource limited settings cannot justify its examination.

There was no literature and this study did not evaluate the cost effectiveness to support the number of sputum sample cultures required to detect one additional PTB case on the second and third sputum culture among HIV infected smear negative PTB suspects hence this study sought for expert opinions. However, the observed number of samples needed to be examined to identify an additional PTB case ranged from 11–30 samples by the second sample culture, and 35–56 samples by the third sputum sample culture. The number of samples by the 3rd sputum culture exceeded the 17 samples suggested by the TB experts as acceptable. Based on these findings, two sputum sample cultures may be optimal. However, it is worth noting that the number of sputum samples required for an additional PTB case depends on the prevalence of TB in a given population and the ability to correctly suspect a TB case. In areas were TB prevalence is lower than in the current study, the number required for the second and third sputum cultures could be higher. This could further reduce the justification for the third and even the second sputum sample culture.

This study findings could add significant information towards the practices especially in Uganda where varying number of sputum samples (1 to 3) are collected for TB culture, as well as to the global policy formulations as it was previously documented to improve access and efficiency through reduced workload which in turn improves quality [15].

We recommend that country’s TB programs adopt two sputum samples for culture, however, a bigger study to look at patient outcomes and cost-effectiveness of our findings may be needed. Furthermore, given the wide variation of the necessary resources and operational costs for TB culture, it is essential to employ appropriate recommendations and policies locally. Settings with enough resources may continue examining the 3^rd^ sputum sample using culture as the IY of 7% may not easily be ignored especially among HIV infected persons where early diagnosis is paramount mainly before ART initiation.

Previous studies were done in general HIV population irrespective of their smear results, and few have looked at the gain from the different sputum samples [2], [3]. This gives our study strength since it was conducted in an HIV infected smear negative TB and applied special analytical techniques to determine the yield of each sputum sample culture.

Previous studies among smear negative patient also did not analyze their results to find out the number of cultures needed to find an additional TB case in a given population considering the first, second and third sputum sample cultures.

### Limitations

We acknowledge the small sample size due to limited resources; but being the first of its kind in the country and in this study population, the findings of this study may be important for studies aiming at larger sample sizes.

This study was also conducted in a research setting with special attention to sample transportation and processing as expected in a clinical research laboratory hence the findings of this study may not reflect the routine. The study analysis considered all contaminated results as negative; this could have underestimated the TB prevalence in the study population.

It is also worth noting that we did not follow those who were negative as per the national TB guidelines. Furthermore, future studies to look at the gain from two serial sputum sample cultures collected on the same day “Front-loading” may be necessary as patients will only make one visit to the health facility and also may increase adherence to sputum collection and efficiency.

## Methods

This study was approved by the Higher Degree Research and Ethics Committee (HDREC) of Makerere University School Public Health, Kampala and the Uganda National Council of Science and Technology (UNCST). All participants provided a written informed consent before participating in this study. The aim of this study was to determine the incremental yield and the number of sputum samples needed to find an additional PTB case by culture on the second and third serial sputum sample among HIV infected smear negative PTB suspects in Kampala, Uganda.

### Hypothesis

Since there was no literature on the number of samples required for an additional PTB case, five international experts in mycobacteriology and/or public health TB control programs, who were not involved in the current study, were requested for their opinion on the number of samples. The maximum number of samples for the second sputum was 12, while for the third was 17 samples.

We therefore hypothesized that not more than 12 sputum cultures by the second and 17 by the third sputum sample are needed to be examined for an additional PTB case with a second and third serial diagnostic sputum culture.

### Study Setting

All participants were HIV positive and managed according to the WHO HIV/AIDS clinical stages with some center having capacity for CD4 counts. Participants were from three HIV care clinics and one general TB ward of Mulago National referral hospital in Kampala, Uganda (Infectious Diseases institute, Makerere- Mbarara Joint Aids Project [MJAP], in patient wards 4A, and general TB ward 5 and 6). In these clinics all PTB suspects are screened basing on clinical signs and symptoms together with chest X-ray and diagnosed by sputum smear microscopy using Ziehl Neelsen (ZN) method.

### Study Design

This was a cross-sectional study in which a total of 203 participants were screened and only 181 enrolled. Those eligible were male and female HIV-infected, at least 18 years and above, with cough for more than two weeks and at least two negative sputum smears and no history of PTB treatment. Participants were instructed to take a deep breath and cough to provide an on spot sputum sample and given another container to provide an early morning sample. On the following day after delivering early morning sputum sample, a third spot sputum sample was provided.

### Laboratory Methods

At the participating clinic laboratories, sputum smear microscopy was performed on these samples using standard ZN method [16]. Following smear microscopy, sputum samples from participants with three negative samples were briefly stored at 2–8°c and transported to the laboratory, in a box with ice packs, for culture.

At the Mycobacteriology (BSL-3) laboratory, smear microscopy was repeated [16]. Participants whose results turned positive on repeat where excluded, however, their smear results were communicated for patient management.

#### Sputum culture

All samples from smear negative participants were cultured according to standard operating procedures. All cultures were incubated till 8 weeks on LJ (Becton and Dickson, Franklin Lakes, NJ USA) [17] and six weeks in the BD BACTEC™ MGIT ™ 960 (Becton and Dickson, Franklin Lakes, NJ USA) system [18]. For identification of *Mycobacterium tuberculosis* complex (MTBC), cultures in which mycobacteria grew were subjected to Capilia TB Neo™ (TAUN, Numazu, Japan) assay as described in elsewhere [19], [20].

A participant’s culture result was considered contaminated if the LJ slants or MGIT cultures from all specimens revealed growth of organisms other than mycobacteria; otherwise, a positive result was taken as at least a single colony on solid media, or a single positive MGIT culture that revealed AFB on ZN microscopy and was positive upon Capilia TB Neo™. The absence of colonies on LJ medium or fluorescence on MGIT was reported as a negative result. All results were entered into a computerized laboratory access database and linked with patient records on the sample reporting form.

#### Quality assurance

Internationally acceptable quality control procedures for TB diagnosis were implemented at each collection site e.g. sputum container quality, proper sputum collection, precautions given to participants to avoid sample contamination at pre-analytical (sputum quantity and quality plus proper labeling).

Analytical quality procedures included; quality control of strains to confirm growth of AFB as well as staining with known positive and negative controls and checking the media quality using control strains (H_37_Rv ATCC 27294; *M. kansasii,* ATCC 12478; *M. fortuitum,* ATCC 6841). We also included artificial sputum (a dummy sterile sample made of eggs and Methylcellulose, Sigma-Adrich MO267) in each batch of 12 samples to rule out cross-contamination. At post-analytical stages of the laboratory procedures, control measures such as proof reading were taken to minimize chances of false-results.

### Data Management

Data were crosschecked on the data collection forms for completeness before entry into a computer in an excel sheet and quality controlled by checking 10% of the entered records to resolve discrepancies. Data were analyzed using Stata SE software, (version 11, Stata Corp LP, College station TX 77849, USA), for descriptive statistics. The unit of analysis was the sample type (spot, morning, spot) and a *p*-value <0.05 was considered significant.

The potential IY and the number of samples required to identify an additional PTB case was analyzed according to Ipuge Y et al (1996) [21] using an already developed excel database that was used in previous studies [7], [22].

The first step involved calculation of the proportion of suspects positive by each of the sputum culture done i.e. 1^st^, 2^nd^ and 3^rd^. **A case** was a person who was positive by any of the sputum cultures i.e. LJ, MGIT or a combination of both.

In a second step, the potential incremental yield from serial sputum culture was calculated.

#### Proportion of suspects positive on the 1^st^, 2^nd^ and 3^rd^ sputum culture

Not all patients managed to submit the required three serial sputum samples; therefore we categorized results into six patterns: **Px, NPx, NNP, NNN, NN9, N99.** Where P was a positive culture result, x a subsequent result of no interest, N a negative result, and 9 a missing result.

The fraction of persons who were found positive on the first sputum culture was denoted as **S_d_1: S_d_1 = Px/(Px+NPx+NNP+NNN+NN9+N99).**

The numerator comprised those suspects who were positive on the first sputum culture, i.e., Px.

The denominator comprised those suspects with at least the first sputum culture done.

The fraction of persons who were found to be negative on the first sputum culture but positive on the second was denoted as S_d_2: **S_d_2 = NPx/(NPx+NNP+NNN+NN9).**

The fraction of those who were found to be positive only on the third sputum culture (negative on the first and second) was denoted as S_d_3: **S_d_3 = NNP/(NNP+NNN).**

To calculate the incremental yield, the assumption made was that those with an N99 result had the same probability of being positive on the second sputum culture as those with an NPx result, and those with a NN9 result the same on the third sputum culture as those with a NNP result. Those (positive) who were missed by failing to do a second sputum culture were denoted as M_d_2 and those (positive) who were missed by failing to do the third sputum culture were denoted as M_d_3, and the total (positive) missed by doing neither the second nor the third was denoted as M_d_.

#### For M_d_2 we obtain: M_d_2 = S_d_2 * N99

Calculating M_d_3 took into account both those who were missed the second and those who missed the third sputum culture:

M_d_3 = S_d_3 * (NN9 = (1 - S_d_2) * N99), then:

M_d_ = M_d_2+M_d_3,

thus, the observed (recorded) number of positive Od:

O_d_ = Px+NPx+NNP and the expected number of positive Ed, calculated from the observed and the missed was: E_d_ = O_d_+M_d._

The expected proportion positive was: R_d_ = E_d_/A_d,_ (_Ad = _Number of suspects).

Next, the fractions for the potential yield from the first (F_d_1), the potential incremental yield on the second (F_d_2), and the third (F_d_3) sputum cultures were calculated:

F_d_1 = Px/E_d, _F_d_2 = (M_d_2+NPx)/E_d, _F_d_3 = (M_d_3+NNP)/E_d._

In the final step, the product of the expected proportion positive (R_d_) was multiplied by each potential incremental fraction (F_d_1, F_d_2 and F_d_3) to give the overall fraction positive on the respective sputum culture.

The reciprocal values of the products of (F_d_2 and F_d_3) gave the number of sputum culture needed to find one (additional) case on the second and on the third sputum culture considering those missed by not doing the second and the third sputum culture.
